# Exploring potential value of neutrophil extracellular traps in major depressive disorder

**DOI:** 10.3389/fimmu.2026.1818375

**Published:** 2026-05-12

**Authors:** Yachen Shi, Gaojia Zhang, Yi Ji, Guangjun Xi, Yiping You, Jingyu Deng, Qianqian Gao, Haixia Mao, Xuefang Lu, Xiaoxuan Zhang, Wei Ji, Xiaohang Wang, Pan Wang, Mengmeng Zhong, Yan Han, Peng Yuan, Xiangming Fang, Feng Wang

**Affiliations:** 1 Department of Neurology, the Affiliated Wuxi People’s Hospital of Nanjing Medical University, Wuxi People’s Hospital, Wuxi Medical Center, Nanjing Medical University, Wuxi, China; 2 Comprehensive Stroke Center, the Affiliated Wuxi People’s Hospital of Nanjing Medical University, Wuxi People’s Hospital, Wuxi Medical Center, Nanjing Medical University, Wuxi, China; 3 Department of Psychology and Sleep Medicine, The Second Hospital of Anhui Medical University, Hefei, China; 4 Department of Radiology, the Affiliated Wuxi People’s Hospital of Nanjing Medical University, Wuxi People’s Hospital, Wuxi Medical Center, Nanjing Medical University, Wuxi, China; 5 Department of Neurology, Huidong People’s Hospital, Huizhou, China; 6 Department of Rehabilitation Medicine, the Affiliated Wuxi People’s Hospital of Nanjing Medical University, Wuxi People’s Hospital, Wuxi Medical Center, Nanjing Medical University, Wuxi, China; 7 Department of Neurosurgery, the Affiliated Wuxi People’s Hospital of Nanjing Medical University, Wuxi People’s Hospital, Wuxi Medical Center, Nanjing Medical University, Wuxi, China; 8 Institute of Translational Medicine, Jiangsu Key Laboratory of Integrated Traditional Chinese and Western Medicine for Prevention and Treatment of Senile Diseases, Medical College, Yangzhou University, Yangzhou, China

**Keywords:** antidepressive treatment, biomarker, inflammatory response, major depressive disorder, neutrophil extracellular traps

## Abstract

**Background:**

Neutrophil extracellular traps (NETs) can induce cellular and tissue damage through inflammatory responses. While the involvement of NETs in psychiatric disorders has shown preliminary potential, a systematic exploration of their link with major depressive disorder (MDD) is imperative. This study evaluated the clinical potential of three NET markers—myeloperoxidase (MPO)-DNA, neutrophil elastase (NE)-DNA, and citrullinated histones (citH3)—for diagnosing MDD and predicting treatment response.

**Methods:**

Two independent clinical cohorts (Cohort 1: n=83; Cohort 2: n=60) and a chronic unpredictable mild stress (CUMS) mouse model were used. Physiotherapy and pharmacotherapy were administered to the two cohorts, respectively. NET markers were measured in plasma samples. Levels of NETs in the hippocampus were detected in CUMS mice. Pharmacological blockade of NET formation was performed in mice.

**Results:**

(1) Independently validated across two cohorts, plasma levels of NET markers were significantly higher in MDD patients than in healthy participants. (2) In MDD patients, plasma NET markers significantly correlated with neuropsychological assessment scores, serum levels of inflammatory indices, and abnormal activation of the right calcarine and cuneus. (3) Relationship between NETs and C-reactive protein has an significant effect on depressive symptoms. (4) These NET markers could predict changes in 24-item Hamilton Depression Rating Scale scores after antidepressive treatments. (5) Compared with controls, CUMS mice exhibited significantly elevated levels of NE and MPO in the hippocampus. Preventing NET formation significantly reduced NE and MPO levels in plasma and hippocampus, and alleviated depressive-like behavior in CUMS mice.

**Conclusions:**

Plasma NETs may be associated with the occurrence and progression of MDD, potentially via inflammatory mechanisms. Plasma NET markers may be used as valuable biomarkers to diagnose MDD and predict patient response to antidepressive treatment. Targeting NET formation could represent a potential therapeutic strategy for depression.

## Introduction

Major depressive disorder (MDD) is one of the leading causes of disability worldwide and represents a significant public health issue (1, 2). It imposes a substantial burden not only on affected individuals, but also on their families, communities, and broader economies (3). The symptoms of MDD include depressed mood, loss of interest, changes in weight or appetite, and an increased likelihood of suicide (4, 5). MDD involves a complex pathological mechanisms. Several hypotheses have been proposed to explain its etiology, including the inflammatory hypothesis, hypothalamic-pituitary-adrenal axis dysfunction hypothesis, monoamine hypothesis, and genetic/epigenetic anomaly hypothesis. (6–9). While no single hypothesis fully accounts for the pathophysiology of MDD, they have collectively informed the development of diagnostic and therapeutic approaches. In terms of diagnosis, inflammatory markers such as C-reactive protein (CRP), interleukin-6 (IL-6), and tumor necrosis factor-alpha (TNF-α) have been consistently identified as potential biomarkers for MDD in multiple meta-analyses (10–12). Meanwhile, neurotrophic factors, notably brain-derived neurotrophic factor (BDNF), also show diagnostic value and may reflect treatment response (13–16). Furthermore, emerging markers, including microRNA, circular RNA, and gut microbiota, also demonstrate potential for clinical application in MDD (17–19). Regarding treatment, pharmacotherapy for MDD commonly includes selective serotonin reuptake inhibitors (SSRIs) and serotonin-norepinephrine reuptake inhibitors (SNRIs) (20). In addition, physiotherapeutic interventions such as repetitive transcranial magnetic stimulation (rTMS) are increasingly used to modulate inflammatory responses and enhance neural plasticity in patients with MDD (21–23). Although international diagnostic guidelines such as the Diagnostic and Statistical Manual of Mental Disorders (DSM-V) (24) provide valuable criteria for clinical diagnosis, the lack of disease-specific symptoms and objective biomarkers for MDD complicates its identification and hinders early prevention in clinical practice.

Neutrophil extracellular traps (NETs) are released by activated neutrophils in response to various stimuli (25). During this process, neutrophils undergo lysis, leading to the extracellular release of myeloperoxidase (MPO)-DNA complexes, neutrophil elastase (NE)-DNA complexes, and citrullinated histones (citH3) (26). Peptidyl arginine deiminase 4 (PAD4) is an enzyme that catalyzes the conversion of arginine to citrulline. It is expressed in neutrophils and, upon activation, promotes the formation of NETs (27). Due to the non-specific actions of these proteins, NETs can trigger uncontrolled inflammatory responses that result in cellular and tissue damage, contributing to diseases such as systemic lupus erythematosus and inflammatory bowel disease (28, 29). Besides, NETs also contribute to increased morbidity and poor prognosis in cardiovascular diseases, such as ischemic stroke, heart failure, and acute myocardial infarction (30, 31). In the field of psychiatry, Corsi-Zuelli et al. first demonstrated elevated levels of NETs in the plasma of patients with early-stage schizophrenia and in adolescent stressed rats compared with controls (32). Additionally, citH3, a marker of NET formation, was significantly increased in the plasma of wild-type mice following lipopolysaccharide (LPS) administration (33). Moreover, LPS-induced depression-like behaviors were markedly attenuated in NET-deficient mice, suggesting a role for peripheral NETs in mediating these behavioral changes (33). Nevertheless, the formation and functional impact of NETs in psychiatric disorders remain incompletely understood. In particular, there is still insufficient evidence to evaluate the potential clinical utility of NETs in MDD.

We aimed to assess plasma NET levels in patients with MDD and analyze their association with depressive symptom severity and serum inflammatory markers through a multicenter design with independent clinical cohorts. Meanwhile, using clinical follow-up data from antidepressant treatment, we also aimed to evaluate the potential of plasma NETs to predict treatment response. In addition, we examined the expression of NETs in the brain and peripheral blood using a classic animal model of depression.

## Method and materials

### Participants

The present study included two independent cohorts. Cohort 1, recruited from the Affiliated Wuxi People’s Hospital of Nanjing Medical University, encompassed 38 adult MDD patients and 45 healthy controls (HCs). Cohort 2, recruited from the Second Affiliated Hospital of Anhui Medical University, consisted of 60 adult MDD patients. The ethical approval was obtained from the Ethics Committee of the Affiliated Wuxi People’s Hospital of Nanjing Medical University (approval number: KY22082). Moreover, this study was also approved by the Ethics Committees of the Second Hospital of Anhui Medical University (approval number: SL-YX2024-022). All participants or their legal guardians provided informed consent.

All patients diagnosed with MDD fulfilled the diagnostic criteria set forth in the DSM-V (24), determined by a professional psychiatrist (Gaojia Zhang) through the standardized structured clinical interview (detailed operations were displayed in Supplementary Materials). The inclusion criteria for MDD patients were delineated as follows: (1) those undergoing their inaugural episode, irrespective of whether they were receiving outpatient or inpatient care; (2) patients in Cohort 1 had no prior exposure to pharmacological interventions, whereas patients in Cohort 2 had ceased all antidepressant treatments for a duration surpassing two weeks before the study’s initiation; (3) individuals devoid of a familial antecedent of psychosis; and (4) for female participants, being neither pregnant nor lactating. Moreover, HCs exhibited no prior history of DSM-V Axis I disorders, mental health concerns, or substantial physical maladies.

Furthermore, participants also met the following exclusion criteria: (1) the presence of cerebrovascular or neurodegenerative disorders, such as stroke, Alzheimer’s disease, or Parkinson’s disease; (2) comorbid psychiatric disorders, such as schizophrenia or bipolar disorder; (3) a history of alcohol or drug abuse or dependence; (4) significant physical illnesses, such as autoimmune diseases, endocrine disorders, or impaired cardiac, hepatic, or renal function; (5) a history of traumatic brain injury; or (6) any form of neoplastic disease.

### Observational follow-up

Each MDD patient in Cohort 1 received a standard four-week course of rTMS treatment in accordance with the 2016 Clinical Guidelines of the Canadian Network for Mood and Anxiety Treatments (CANMAT) for adults with major depressive disorder (34, 35). The treatment was administered using a Magstim Rapid stimulator with a figure-of-eight coil (Magstim Company Ltd, UK). The left dorsolateral prefrontal cortex as the stimulation site was identified using the “5-cm rule” (36). The resting motor threshold (RMT) was determined as the lowest stimulus intensity that produced a motor evoked potential in the right abductor pollicis brevis muscle in at least 50% of trials, following single-pulse TMS applied to the primary motor cortex. Stimulation parameters were set at 10 Hz and 110% of RMT, with trains of 4 seconds on and 26 seconds off, delivering 3,000 pulses per day over five days per week. During the treatment period, no patients received any psychotropic medication.

Furthermore, all patients with MDD in Cohort 2 underwent a four-week clinical antidepressant treatment. As treatment preferences varied among the attending physicians, these patients did not receive identical therapeutic regimens. Nonetheless, during the treatment period, only one type of SSRI or SNRI was used as the antidepressant (see details in Supplementary Materials). All enrolled patients were supervised by their family members during daily medication administration and no instances of missed doses were recorded throughout the follow-up period.

Notably, due to the primary objective of this clinical study was not to assess the efficacy of antidepressive treatment, the study was designed as an observational follow-up rather than a randomized controlled trial. The present study did not intervene the established therapeutic schedule of clinicians for each MDD patients.

### Neuropsychological assessments

The neuropsychological assessments for each participant were administered by a trained psychiatrist. Assessments were conducted for HCs at baseline and for patients with MDD both before and after treatment.

In Cohort 1, the 24-item Hamilton Depression Rating Scale (HAMD-24) and the Self-Rating Depression Scale (SDS) were used to assess depressive symptoms. The Hamilton Anxiety Rating Scale (HAMA) and the Self-Rating Anxiety Scale (SAS) were employed to assess anxiety symptoms. The Snaith-Hamilton Pleasure Scale (SHAPS) was administered to measure anhedonia in these participants. In Cohort 2, the HAMD-24, SDS, and SAS were utilized to evaluate psychological status.

### Collection of blood samples

Between 8:00 a.m. and 9:00 a.m., fasting peripheral venous blood specimens were drawn from each participant. Two distinct types of vacutainer tubes were utilized: one was a plain tube devoid of any anticoagulant, and the other was an EDTA-coated tube. Blood samples were collected from HCs at baseline and from patients with MDD before and after the initiation of treatment.

For serum preparation, blood samples collected in plain vacutainer tubes (without anticoagulant) were promptly centrifuged at 3,500 revolutions per minute for 10 minutes at 4 °C within 30 minutes of collection. The resulting serum was then divided into aliquots and stored at -80 °C until further analysis. Additionally, within the same 30-minute window post-collection, blood samples in EDTA-coated tubes were centrifuged at 1,000 × g at 4 °C for 10 minutes. Following centrifugation, the plasma was carefully aspirated and stored at -80 °C until required for subsequent use.

### Enzyme-linked Immunosorbent Assay measurement

All blood samples from both cohorts underwent ELISA testing on the same day in the laboratory of Wuxi People’s Hospital. The plasma concentrations of three circulating NET markers-namely NE-DNA, MPO-DNA, and citH3-were measured using commercial ELISA kits (Hengyuan Biotech Co., Ltd, Shanghai, China). Additionally, serum levels of BDNF, CRP, IL-6, and TNF-α were evaluated with corresponding ELISA kits (FineTest, Wuhan, China: BDNF, Cat. No. EH0043; IL-6, Cat. No. EH0201; TNF-α, Cat. No. EH0302; CRP, Cat. No. DCRP00, R&D Systems, Minneapolis, MN, USA) following the manufacturers’ instructions. All samples were analyzed in triplicate. Protein concentrations were calculated from standard curves. The intra- and inter-assay coefficients of variation were both below 5%. Meanwhile, plasma samples were used to measure the levels of aspartate aminotransferase (AST) and alanine aminotransferase (ALT) using a conventional automated analyzer (Mitsubishi MEDIENCE, Kobe, Japan).

### MRI data acquisition and preprocessing

Each participant of Cohort 1 received a functional brain MRI using a 3.0-T MR scanner (MAGNETOM Prisma, Siemens Healthcare, Erlangen, Germany). Data preprocessing was conducted using the Data Processing Assistant for RestingState fMRI (DPARSFA 2.3) toolbox. The fractional amplitude of low-frequency fluctuations (fALFF) reflects the strength of regional slow-wave brain activity (37). The mean fALFF values in the regions that significantly different between the MDD and HC groups were extracted (Alphasim multiple comparison correction-adjusted p <no><</no> 0.05). Age, sex, body mass index, and years of education as covariates were adjusted. More methodological details are displayed in the Supplementary Materials.

### Animals

Adult male C57BL/6J mice (6–8 weeks old, 24.0-26.0 g) were purchased from Aniphe Biolaboratory Inc. (Nanjing, China). Prior to experimental procedures, the mice were randomly assigned to different groups and housed in cages under a 12−hour light/12−hour dark cycle (lights on at 07:00). Food and water were available ad libitum. Mice in the chronic unpredictable mild stress (CUMS) group were housed individually, whereas control mice were group-housed. All mice procedures were approved by the Institutional Animal Committee of the the Affiliated Wuxi People’s Hospital of Nanjing Medical University (approval number: DL2023146). All animal procedures were performed in accordance with the National Institutes of Health’s Guide for the Care and Use of Laboratory Animals.

### CUMS protocol and behavioural tests

The mice were subjected to a variety of randomly assigned, low-intensity social and environmental stressors twice daily over a four-week period (38). The stressor protocols included: (1) 24-hour food or water deprivation; (2) overnight illumination; (3) 24-hour removal of cage bedding; (4) 24-hour exposure to dampened bedding; (5) forced swimming in 8 °C water for 5 minutes; (6) tail nip (1 cm from the tail tip); (7) 6-hour physical restraint; and (8) 45°cage tilt for 3 hours.

All behavioral tests were performed in a quiet, low-stimulus environment and scored by a single researcher. Mice were acclimated to the testing room for at least 3 hours prior to evaluation. Test sessions were video-recorded for subsequent analysis. The sucrose preference test and open field test were employed to evaluate the successful induction of the CUMS model and to assess depressive-like behaviors in the animals. Detailed methodologies for the behavioral tests are provided in the Supplementary Materials.

### Drug intervention

In this study, randomly selected CUMS mice received a 14-day drug intervention via tail vein injection, with drugs administered every other day. Three subgroups were treated with three different agents. To degrade NETs, DNase I (11284932001, Roche, Shanghai, China) was injected at a dose of 2.5 mg/kg, and PAD4 activity was inhibited using GSK-199 (17489, Cayman Chemical, Ann Arbor, Michigan, USA) at 3.0 mg/kg (39, 40). The sham group received injections of normal saline (NS). These groups were accordingly designated as the DNase I, GSK-199, and NS groups. Following the 14-day intervention, all mice underwent the open field test.

### Immunofluorescence imaging analysis

The mice were deeply anesthetized by intraperitoneal injection of sodium pentobarbital. Following anesthesia, the brains were rapidly removed and embedded in optimal cutting temperature compound. Hippocampal-containing tissues were sectioned at a thickness of 30 μm using a cryostat. For immunofluorescence staining, sections were incubated with primary anti-MPO (1:50, ab25989, Abcam, Boston, MA, USA), anti-NE (1:500, ab314916, Abcam, Boston, MA, USA), and anti-Iba1 (1:250, 019-19741, Wako Pure Chemicals, Osaka, Japan). Nuclei were counterstained with DAPI (Servicebio, Wuhan, China). Images were acquired using a a fluorescence/confocal slide scanner. Further methodological details are provided in the Supplementary Materials.

### Pro-inflammatory cytokines measurement

The hippocampal tissue was rinsed and homogenized, followed by three freeze-thaw cycles to disrupt the cell membranes. The supernatant was then collected by centrifugation at 5,000 × g for 5 minutes at 4 °C. Levels of the cytokines IL-1β and IL-6 were measured using commercial ELISA kits (Mouse IL-1β, JEB-12787; Mouse IL-6, JEB-12267) according to the manufacturer’s instructions. These kits were purchased from Nanjing Jin Yibai Biological Technology Co., Ltd.

### Plasma collection and measurement

Mice blood samples were collected via orbital puncture using EDTA-anticoagulant tubes prior to anesthesia. Following centrifugation under the same conditions as applied for human blood samples, plasma was harvested. All three markers, NE-DNA, MPO-DNA, and citH3, were subsequently quantified in plasma by ELISA kits (Hengyuan Biotech Co., Ltd, Shanghai, China).

### Statistical analysis

Data analysis was conducted using SPSS version 22.0 (SPSS, Inc., Chicago, IL, USA). Normality of the data was evaluated with the Kolmogorov-Smirnov test. Categorical variables were analyzed using the chi-square test. For continuous variables, the independent-samples t-test was applied for normally distributed data, while the Mann-Whitney U test was used for non-normally distributed data. Changes in variables before and after treatment were compared with the paired t-test. Bonferroni correction was used to conduct the multiple comparison corrections. A one-way ANOVA was used to analyze differences among the three groups, followed by *post hoc* analysis with Bonferroni correction. Receiver operating characteristic (ROC) curves were generated to calculate the area under the curve (AUC), evaluating the diagnostic power of NET markers in distinguishing MDD patients from HCs. The Youden index was applied to determine optimal sensitivity and specificity values. In MDD patients, partial correlation analysis was performed to examine the associations between variables, with adjustments for some factors such as age, sex, years of education, body mass index (BMI), and disease duration. Additionally, linear regression models were constructed to further assess multivariate effects on neuropsychological assessments. The rate of change in assessment scores was calculated as (pre-treatment score – post-treatment score)/pre-treatment score. The absolute change in marker levels was defined as (pre-treatment level – post-treatment level). Statistical significance was set at a two-tailed p-value < 0.05.

## Results

### Analysis of NET markers in participants in cohort 1

#### Comparison of plasma levels of NET markers between patients with MDD and HCs

As shown in **Table 1**, there were no significant differences in age, sex, years of education, BMI, and plasma levels of AST and ALT between patients with MDD and HCs. In addition, MDD patients exhibited significantly higher scores on the HAMD-24, HAMA, SDS, SAS, and SHAPS scales compared to HCs (**Table 1**). Concurrently, serum levels of BDNF, CRP, IL-6, and TNF-α were also significantly elevated in MDD patients relative to HCs (**Table 1**).

**Table 1:** Clinical characteristics of all participants in Cohort 1.

Features	HC (n = 45)	MDD (n = 38)	P-value
Age	33.31 ± 7.41	34.63 ± 8.73	0.319^a^
Sex (female/male)	27/18	23/13	0.128^b^
Education years	16.56 ± 2.62	15.63 ± 2.16	0.106^a^
BMI	22.83 ± 2.93	22.68 ± 2.96	0.569^a^
Plasma AST (U/L)	18.62 ± 6.08	20.68 ± 6.17	0.130^a^
Plasma ALT (U/L)	17.47 ± 5.80	15.63 ± 4.75	0.123^a^
Before treatment			
HAMD-24 scores	2.89 ± 1.91	27.71 ± 5.61	< 0.001^c^
HAMA scores	1.73 ± 1.51	17.26 ± 5.70	< 0.001^c^
SDS scores	37.33 ± 8.79	65.79 ± 8.80	< 0.001^a^
SAS scores	35.89 ± 8.79	56.32 ± 9.92	< 0.001^a^
SHAPS scores	15.07 ± 1.94	25.76 ± 8.62	< 0.001^c^
Serum BDNF (ng/ml)	28.91 ± 7.75	21.36 ± 4.71	< 0.001^a^
Serum CRP (mg/L)	3.17 ± 0.93	4.09 ± 1.02	< 0.001^a^
Serum IL-6 (pg/ml)	34.26 ± 10.16	39.53 ± 11.34	0.028^a^
Serum TNF-α (pg/ml)	41.94 ± 10.37	48.76 ± 13.69	0.012^a^
After treatment			
HAMD-24 scores	–	15.84 ± 5.65	< 0.001^d^
HAMA scores	–	9.47 ± 4.17	< 0.001^d^
SDS scores	–	51.18 ± 10.20	< 0.001^d^
SAS scores	–	45.39 ± 11.24	< 0.001^d^
SHAPS scores	–	22.37 ± 7.48	0.019^d^
Serum BDNF (ng/ml)	–	25.78 ± 5.36	< 0.001^d^
Serum CRP (mg/L)	–	3.58 ± 0.85	0.029^d^
Serum IL-6 (pg/ml)	–	38.14 ± 10.40	0.085^d^
Serum TNF-α (pg/ml)	–	44.93 ± 10.63	0.033^d^

MDD, major depressive disorder; HC, healthy control; BMI, body mass index; HAMD-24, 24-item Hamilton Depression Scale; HAMA, Hamilton anxiety scale; SDS, Self-Rating Depression Scale; SAS, Self-Rating Anxiety Scale; SHAPS, Snaith-Hamilton Pleasure Scale; BDNF, brain-derived neurotrophic factor; CRP, C-reactive protein; IL-6, interleukin-6; TNF-α: tumour necrosis factor alpha; AST, aspartate aminotransferase; ALT, alanine aminotransferase.

^a^ P-values were obtained by Independent-Samples T test.

^b^ P-values were obtained by Mann-Whitney U test.

^c^ P-values were obtained by Chi-square test.

^d^ P-values were obtained by paired sample test between before and after treatment in MDD patients.

**Figure 1:**
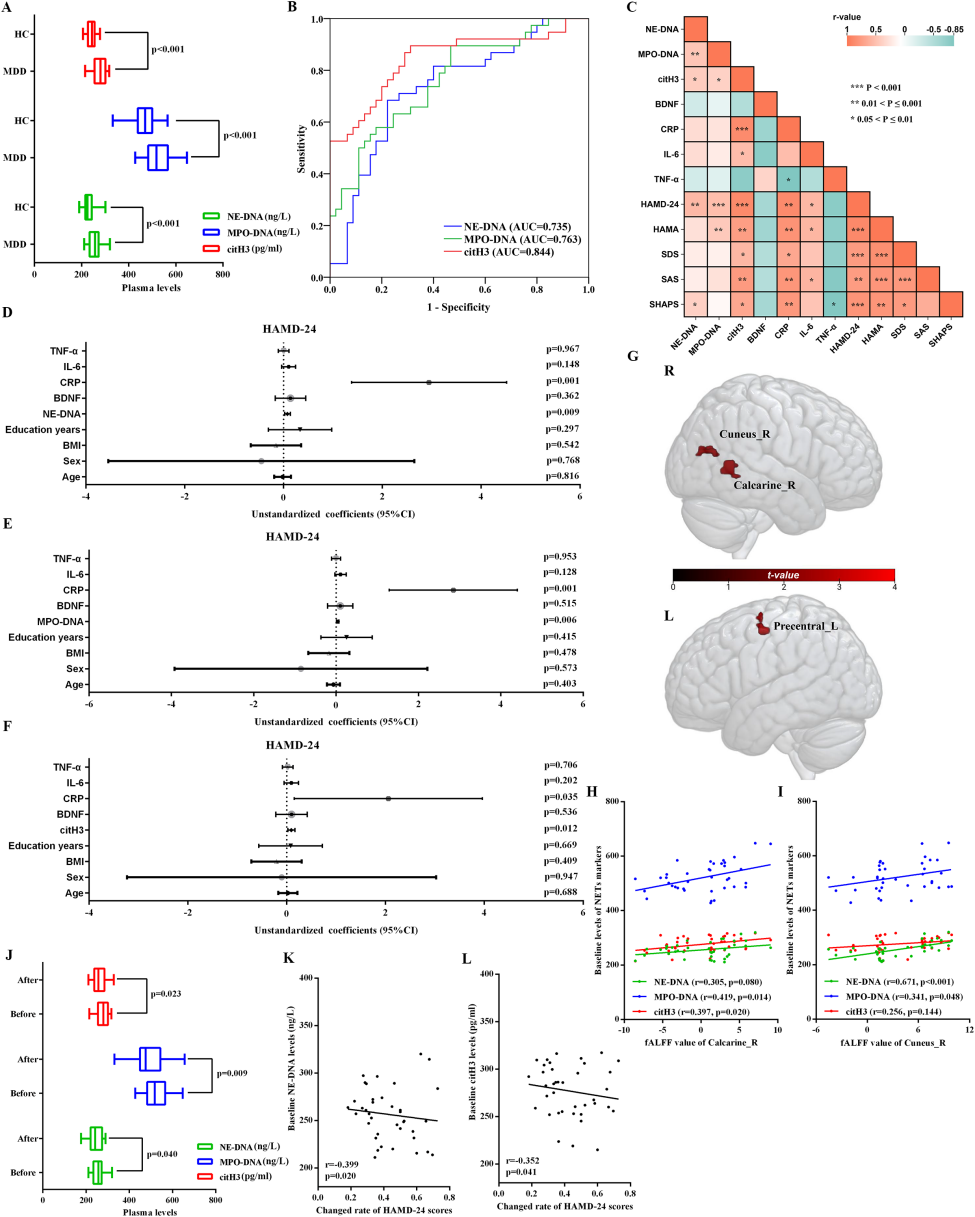
Analysis of plasma NET markers in independent cohort 1. **(A)** Comparison of plasma levels of three NET markers between MDD and HC groups. **(B)** ROC curve analysis of plasma NET markers for identifying MDD patients from HCs. **(C)** Correlation analyses between plasma NET markers and clinical features (including serum levels of BDNF and inflammatory markers and psychological assessment scores) in MDD patients. Age, sex, body mass index, and education years were controlled. **(D-F)** Multivariate analyses for HAMD-24 scores in MDD patients using NE-DNA, MPO-DNF, and citH3, respectively. Age, sex, body mass index, and education years were controlled. **(G)** Three brain regions displayed significantly increased fALFF in 38 MDD patients compared with 45 HCs (p < 0.05, Alphasim multiple comparison correction, voxel number > 18). Covariates were age, sex, body mass index, and education years. **(H-I)** Correlation analyses between plasma levels of three NET markers and fALFF values of the right calcarine and cuneus in MDD patients. Age, sex, body mass index, and education years were controlled. **(J)** Comparison of plasma NETs marker levels before and after treatment in patients with MDD. **(K-L)** Correlation between rates of change of HAMD-24 scores and baseline plasma NE-DNA/citH3 levels in MDD patients with rTMS treatment. Covariates were age, sex, body mass index, and education years. MDD, major depressive disorder; NETs, neutrophil extracellular traps, citH3, citrullinated histones; HAMA, Hamilton anxiety scale; SDS, Self-Rating Depression Scale; SAS, Self-Rating Anxiety Scale; SHAPS, Snaith-Hamilton Pleasure Scale; BDNF, brain-derived neurotrophic factor; CRP, C-reactive protein; IL-6, interleukin-6; TNF-α, tumour necrosis factor alpha; fALFF, fractional amplitude of low-frequency fluctuations; rTMS, repetitive transcranial magnetic stimulation.

Furthermore, plasma levels of NET markers, specifically NE-DNA, MPO-DNA, and citH3, were significantly higher in MDD patients than in HCs (**Figure 1A**). These results met statistical significance of multiple comparisons (p < 0.0166, Bonferroni correction).

#### ROC curves analysis

**Figure 1B** presents the AUC values for plasma NE-DNA, MPO-DNA, and citH3 in distinguishing MDD patients from HCs, which were 0.735, 0.763, and 0.844, respectively. The corresponding specificity and sensitivity for each marker are showed in **Supplementary Table 1**.

#### Association analyses of NET markers in patients with MDD

In **Figure 1C**, a partial correlation matrix was generated to evaluate the relationships between plasma levels of NET markers and neuropsychological assessment scores as well as serum indices in MDD patients prior to treatment, with adjustments for age, sex, education years, and BMI. Plasma citH3 levels exhibited significant positive correlations with HAMD-24, HAMA, SDS, SAS, and SHAPS scores, along with serum CRP and IL-6 levels (**Figure 1C** and **Supplementary Table 2**). Additionally, NE-DNA levels in plasma were significantly correlated with HAMD-24 and SHAPS scores (**Figure 1C** and **Supplementary Table 2**). Likewise, plasma MPO-DNA levels showed significant associations with HAMD-24 and HAMA scores (**Figure 1C** and **Supplementary Table 2**).

Further multivariate regression analysis demonstrated that connection between plasma NET markers and several serum indices exerted significant independent effects on HAMD-24 scores in MDD patients, after adjustment for potential confounders including age, sex, education years, and BMI (**Figures 1D–F**). These findings suggest that a relationship between serum CRP and each of the three plasma NET markers may be associated with HAMD-24 scores. However, the relatively small regression coefficients for the NET markers suggest a potential limitation, potentially reducing the effect sizes for this relationship.

#### Brain fMRI analysis

Compared with the 45 HCs, the 38 MDD patients exhibited significantly increased fALFF values in the right calcarine, right cuneus, and left precentral gyrus (**Figure 1G**). However, only the fALFF values in the right calcarine and cuneus of MDD patients showed significant correlations with HAMD-24 and HAMA scores (**Supplementary Figure 1**). Furthermore, fALFF values in the right calcarine of MDD patients showed significant correlations with plasma levels of all three NET markers (**Figure 1H**). Additionally, a significantly positive correlation was also observed in MDD patients between fALFF values in the right cuneus and plasma levels of NE-DNA and MPO-DNA (**Figure 1I**).

#### Effect of rTMS treatment for NET markers

Following rTMS treatment, MDD patients exhibited significant reductions in all neuropsychological assessment scores compared to pre-treatment levels (**Table 1**). Additionally, serum levels of BDNF, CRP, and TNF-α were significantly lower after treatment than before treatment (**Table 1**). Particularly, plasma levels of all three NET markers were also significantly reduced in MDD patients after rTMS treatment (**Figure 1J**).

The HAMD-24 score change rate was calculated to assess the effect of antidepressant treatment in patients with MDD (41, 42). In MDD patients receiving rTMS treatment, significant correlations were found between the change in serum BDNF levels and the change rates in HAMD-24 and HAMA scores, suggesting that rTMS was effective and improved depression-related clinical and microphenotypes (**Supplementary Figure 2**). Additionally, baseline plasma levels of NE-DNA and citH3 were significantly correlated with the post-treatment change rate in HAMD-24 scores (**Figures 1K, L**), indicating that these baseline levels may predict the efficacy of rTMS as an antidepressant treatment. Age, sex, education years, and BMI as covariates were controlled for these analyses.

### Analysis of NET markers in participants in Cohort 2

#### Expression of NET markers in the plasma

In patients with MDD from Cohort 2, plasma levels of all three NETs were significantly higher than in HCs from Cohort 1, but did not differ significantly from MDD patients in Cohort 1 (of note, no local healthy control cohort was available for Cohort 2). These findings indicate that elevated plasma NET markers are consistently observed in MDD patients across independent cohorts.

#### Association analyses of NET markers

**Figure 2:**
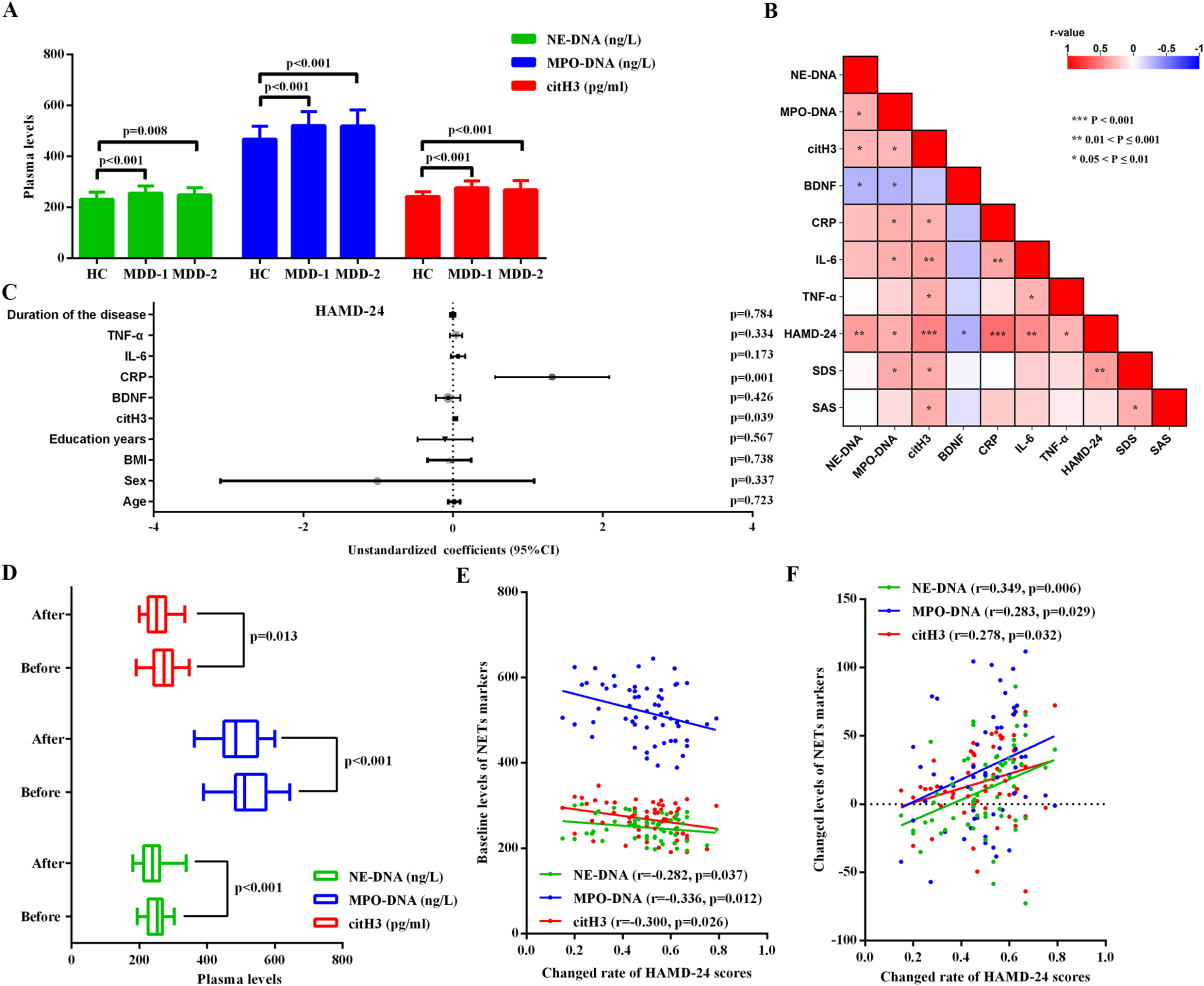
Analysis of plasma NET markers in independent cohort 2. **(A)** Comparison of plasma levels of three NET markers among MDD patients of cohort 1 (MDD1), MDD patients of cohort 2 (MDD2), and HCs (Bonferroni correction for ANOVA test). **(B)** Correlation analyses between plasma NET markers and clinical features (including serum levels of BDNF and inflammatory markers and psychological assessment scores) in MDD patients. Age, sex, body mass index, duration of the disease, and education years were controlled. **(C)** Multivariate analyses for HAMD-24 scores in MDD patients using citH3. Age, sex, body mass index, duration of the disease, and education years were controlled. **(D)** Comparison of plasma NETs marker levels before and after treatment in patients with MDD. **(E)** Correlation between rates of change of HAMD-24 scores and baseline plasma NETs levels in MDD patients with antidepressant drug treatment. Age, sex, body mass index, duration of the disease, and education years were adjusted. **(F)** Correlation between rates of change of HAMD-24 scores and changed value of plasma NETs levels in MDD patients after the antidepressant drug treatment. Age, sex, body mass index, duration of the disease, and education years were adjusted. MDD, major depressive disorder; NETs, neutrophil extracellular traps, MPO, myeloperoxidase; NE, neutrophil elastase; citH3, citrullinated histones; HAMD-24, 24-item Hamilton Depression Scale; SDS, Self-Rating Depression Scale; SAS, Self-Rating Anxiety Scale; BDNF, brain-derived neurotrophic factor; CRP, C-reactive protein; IL-6, interleukin-6; TNF-α, tumour necrosis factor alpha; fALFF, fractional amplitude of low-frequency fluctuations.

A partial correlation matrix, controlling for age, sex, BMI, illness duration, and years of education, is presented in **Figure 2B** to illustrate the association findings. The results revealed significant positive correlations between HAMD-24 scores and plasma levels of all three NET markers (**Figure 2B** and **Supplementary Table 3**). SDS scores were positively correlated with plasma levels of MPO-DNA and citH3, while SAS scores were positively correlated only with citH3 levels (**Figure 2B** and **Supplementary Table 3**). Furthermore, serum levels of CRP, IL-6, and TNF-α were positively correlated with plasma citH3 (**Figure 2B** and **Supplementary Table 3**). Plasma MPO-DNA levels showed significant positive correlations with serum CRP and IL-6 (**Figure 2B** and **Supplementary Table 3**). In contrast, serum BDNF levels were negatively correlated with plasma levels of NE-DNA and MPO-DNA (**Figure 2B** and **Supplementary Table 3**).

Multivariate regression analysis further indicated that the underlying association between plasma citH3 and serum CRP have an significant independent effect on HAMD-24 scores in patients with MDD, even after adjusting for age, sex, BMI, illness duration, and education years (**Figure 2C**). Nevertheless, the relatively small regression coefficients for the NET markers may reduce the effect sizes for this relationship.

#### Effects of NETs marker for antidepressant treatment

Following antidepressant treatment, 60 patients with MDD exhibited significant reductions in HAMD-24, SDS, and SAS scores (**Table 2**). The plasma NET markers also showed a significant reduction by the end of treatment compared with baseline levels (**Figure 2D**). Additionally, the rate of change in HAMD-24 scores was calculated between the pre- and post-treatment time points. Correlation analysis showed a significant association between this rate of change and the baseline plasma levels of all three NET markers (**Figure 2E**). Meanwhile, the rate of change in HAMD-24 scores was significantly correlated with the change in plasma levels of NET markers (**Figure 2F**). All analyses were adjusted for age, sex, BMI, illness duration, and education years.

**Table 2:** Clinical characteristics of 60 MDD patients in Cohort 2.

Features	Before treatment	After treatment	P-value
Age	28.13 ± 11.26	–	–
Sex (female/male)	41/19	–	–
Education years	11.96 ± 2.57	–	–
BMI	22.59 ± 3.29	–	–
Duration of the disease, month	27.25 ± 26.80	–	–
HAMD-24 scores	20.20 ± 4.25	10.37 ± 4.02	< 0.001^a^
SDS scores	70.53 ± 9.94	51.27 ± 12.18	< 0.001^a^
SAS scores	59.65 ± 12.90	48.35 ± 13.60	< 0.001^a^
Serum BDNF (ng/ml)	20.92 ± 5.82	–	–
Serum CRP (mg/L)	4.70 ± 1.30	–	–
Serum IL-6 (pg/ml)	38.89 ± 10.61	–	–
Serum TNF-α (pg/ml)	45.54 ± 12.44	–	–
SSRIs or SNRIs, N%			
Escitalopram	25 (41.67%)	–	–
Sertraline	10 (16.67%)	–	–
Duloxetine	14 (23.33%)	–	–
Fluvoxamine	5 (8.33%)	–	–
Paroxetine	6 (10.00%)	–	–

MDD, major depressive disorder; BMI, body mass index; HAMD-24, 24-item Hamilton Depression Scale; SDS, Self-Rating Depression Scale; SAS, Self-Rating Anxiety Scale; BDNF, brain-derived neurotrophic factor; CRP, C-reactive protein; IL-6, interleukin-6; TNF-α, tumour necrosis factor alpha; SSRIs, selective serotonin reuptake inhibitors; SNRIs, serotonin-norepinephrine reuptake inhibitors.

^a^ P-values were obtained by paired sample test.

### Analysis of NET markers in the mice with depressive-like behavior

**Figure 3:**
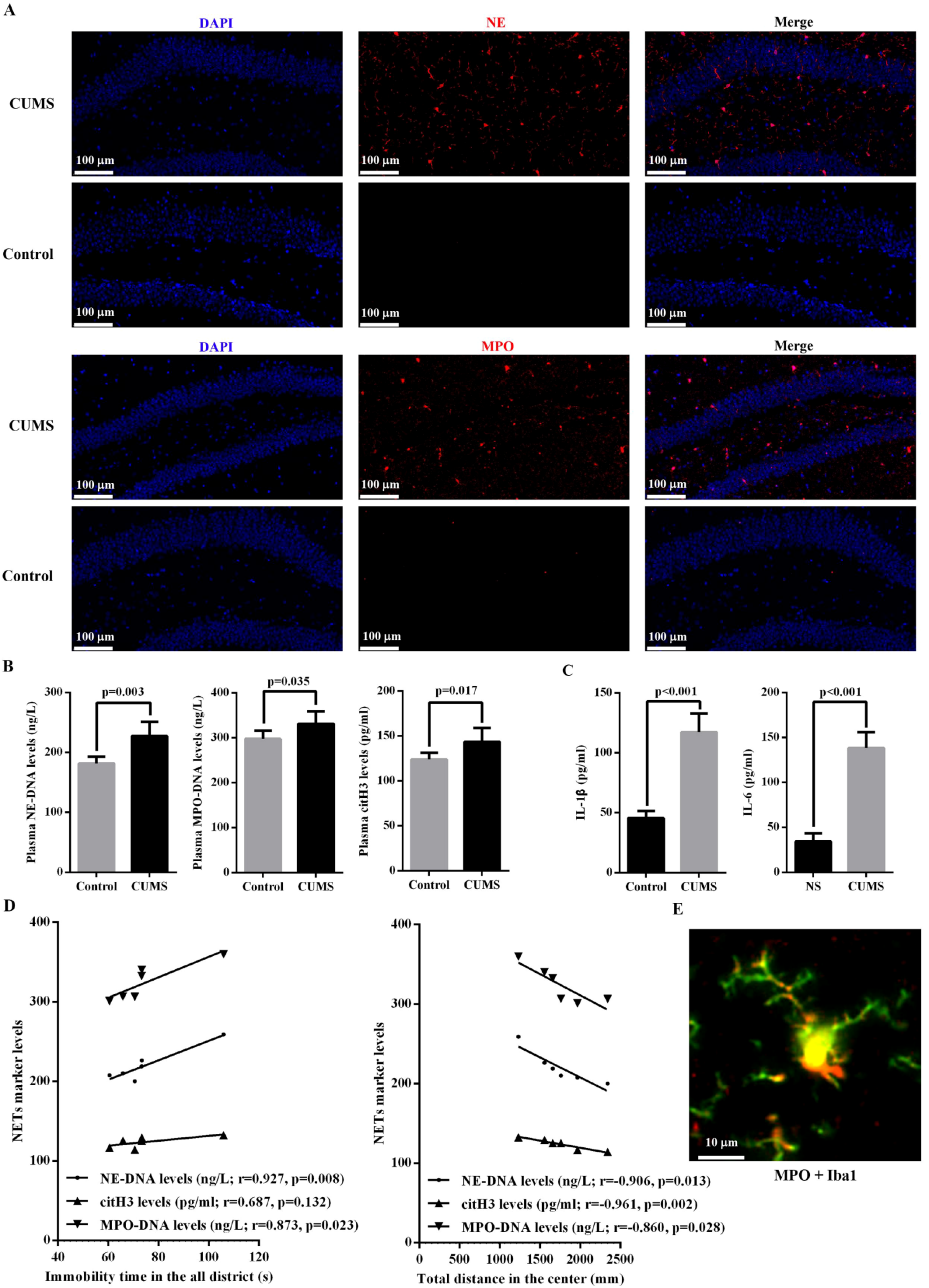
Analysis of plasma NET markers in animal models. **(A)** The distribution of NE and MPO protein in hippocampal regions of CUMS mice and control mice was analyzed by immunohistochemistry (scale: 100μm). **(B)** Comparison of the plasma levels of NE-DNA, MPO-DNA, citH3 between CUMS mice and control mice. **(C)** Comparison of the levels of IL-1β and IL-6 in hippocampal tissues between CUMS mice and control mice. **(D)** Correlation analyses of the plasma levels of NE-DNA, MPO-DNA, citH3 with the specific behavioral indices from the open field test. **(E)** Association between MPO and microglial cell in CUMS animal models (scale: 10μm). NETs, neutrophil extracellular traps; MPO, myeloperoxidase; NE, neutrophil elastase; citH3, citrullinated histones; CUMS, chronic unpredictable mild stress; IL, interleukin.

To investigate whether NET formation is induced during the development of depression, we successfully established a CUMS mouse model (n = 6). Behavioral tests confirmed that CUMS mice performed significantly worse than control mice (**Supplementary Table 4**). In the hippocampal region, the expression of both NE and MPO was significantly upregulated in CUMS mice compared with the control group (**Figure 3A**). Furthermore, plasma levels of NE-DNA, MPO-DNA, and citH3 were significantly higher in CUMS mice than in controls (**Figure 3B**). Moreover, CUMS mice exhibited significantly increased levels of IL-1β and IL-6 in hippocampal tissues compared with controls (**Figure 3C**). Correlation analyses further indicated significant associations between plasma levels of these NET markers and specific behavioral indices from the open field test, including immobility time in the all district and distance traveled in the center (**Figure 3D**). In addition, co-localization of NE/MPO and immunofluorescent markers for microglia (Iba-1) in the hippocampus of the mice supported that these NETs-associated proteins successfully targeted to microglia (**Figure 3E** and **Supplementary Figure 3**).

**Figure 4:**
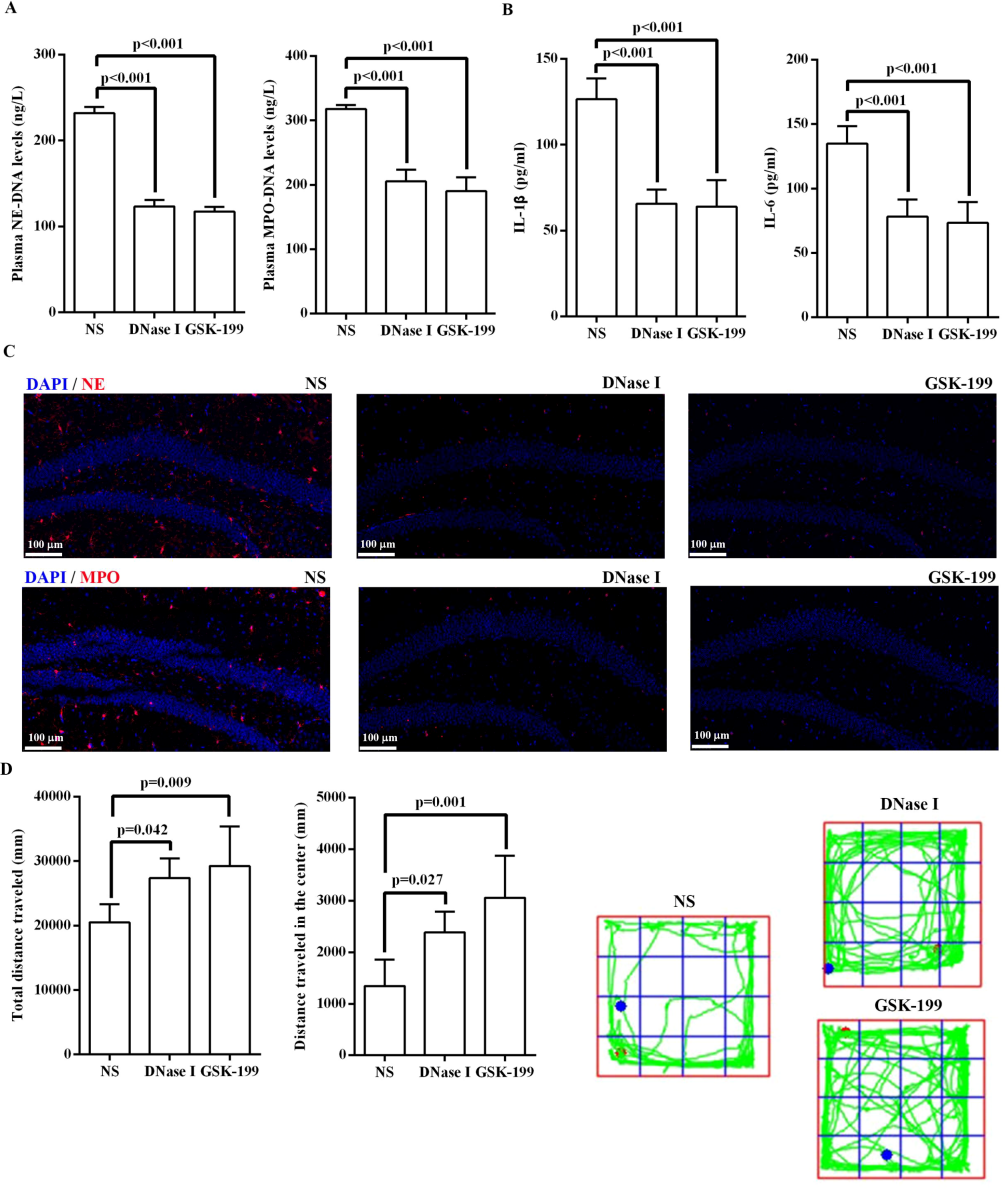
Influence of blocking NET formations in CUMS mice. **(A)** Comparison of plasma levels of NETs among three intervention group. **(B)** Comparison of the levels of IL-1β and IL-6 in hippocampal tissues among three intervention group. **(C)** The distribution of NE and MPO protein in hippocampal regions of CUMS mice among three intervention group (scale: 100μm). **(D)** Comparison of behavioral outcomes among three intervention group. NETs, neutrophil extracellular traps; CUMS, chronic unpredictable mild stress; MPO, myeloperoxidase; NE, neutrophil elastase; NS, normal saline; IL, interleukin.

To confirm the role of NETs in the development of MDD, we employed multiple strategies to target NETs in the CUMS mouse model. Mice were divided into three groups (n = 6 per group) and received injections of NS, DNase I, and GSK-199, respectively. Degradation of NETs with DNase I significantly reduced the plasma levels of NE and MPO compared with the NS group (**Figure 4A**). Compared with the NS group, DNase I-treated mice exhibited significantly reduced hippocampal expression of IL-1β and IL-6, markedly downregulated levels of NE and MPO in the hippocampal region, and significantly improved behavioral performance (**Figures 4B–D**). Furthermore, the specific PAD4 inhibitor GSK-199 was used to affect the role of PAD4 on mediating NET formation (43). Similarly, administration of GSK-199 significantly decreased plasma NE and MPO levels and reduced NET expression in the hippocampus of CUMS mice relative to the NS group (**Figure 4A, C**). Moreover, GSK-199-treated mice exhibited significantly decreased levels of IL-1β and IL-6 in the hippocampus and improved behavioral outcomes compared to NS-treated CUMS mice (**Figure 4B, D**).

## Discussion

The main findings of the current study are as follows. (1) Plasma levels of NET markers (NE-DNA, MPO-DNA, and citH3) were significantly higher in MDD patients than in HCs in the primary cohort. A similar trend was observed in a second independent patient cohort, although no local HCs were available for comparison. (2) Significant correlations were observed between plasma NET markers and neuropsychological assessment scores, as well as serum indices, in MDD patients from two independent cohorts. (3) CRP, as an indicator of potential inflammatory response, may be associated with the effect of plasma NETs on depressive symptoms in MDD patients, a finding corroborated by two independent cohorts. (4) Plasma NET markers may be significantly associated with abnormal activation of the right calcarine and cuneus in MDD patients. (5) Antidepressant treatments, including both physiotherapy and pharmacotherapy, could decrease the expression of plasma NET markers in patients with MDD, and these markers may have predictive value for therapeutic outcomes. (6) Compared with control mice, CUMS mice exhibited significantly elevated levels of NE and MPO in the hippocampus, along with significantly increased plasma levels of NE-DNA, MPO-DNA, and citH3. (7) Microglia may be activated through the uptake of NE and MPO in the hippocampus. (8) Inhibition of NET formation could reduce NET levels in the peripheral blood and hippocampus and improve depressive-like behavior in CUMS mice. Therefore, plasma NETs may be associated with the occurrence and progression of MDD, potentially through mechanisms involving inflammatory activation. Notably, plasma NETs could serve as potential biomarkers for identifying patients with MDD and predicting their response to antidepressive treatment. Targeting dysregulated NET formation could improve depressive behaviors, offering a novel therapeutic insight for MDD.

The present study possesses several notable strengths. First, independent multi-center validation provided robust evidence supporting the association between plasma NET levels and depressive symptoms in MDD patients. Second, follow-up data from clinical interventions offered new insights into the potential utility of plasma NET levels in predicting responses to various antidepressant treatments. Third, rigorous statistical analyses were employed, significantly enhancing the reliability of the findings. Fourth, the study employed macroscopic, mesoscopic, and microscopic approaches, providing robust evidence to support the potential association between peripheral NETs and MDD. Fifth, the use of a CUMS animal model effectively recapitulates chronic pathological changes relevant to MDD, offering substantial face validity for modeling core depressive symptoms observed in MDD patients (44).

Similar to a previous study in schizophrenia (32), the present study found that plasma levels of NE-DNA, MPO-DNA, and citH3 were significantly higher in MDD patients than in HCs in the primary cohort. A similar trend was observed in an independent patient cohort, although interpretation is limited by the absence of matched HCs. These NET markers, particularly citH3, demonstrated potentially diagnostic performance in distinguishing MDD patients from HCs. Since elevated levels of NET markers are also present in other diseases (30–32), it is not yet possible to consider NETs as specific markers for MDD. In fact, we regard NETs as potential candidates for inclusion in a specific diagnostic panel for MDD. In addition, we observed significant correlations between elevated plasma NET markers and depressive assessment scores, suggesting that these plasma NET markers could serve as useful biomarkers reflecting the severity of depressive mood, anhedonia, and anxiety in MDD patients. The collected demographic data and other potential factors that might influence the course of MDD or depressive assessment as covariates were included in the current correlation analyses. However, given the complexity of MDD, future studies should incorporate more potential factors as covariates in relevant analyses, such as assessments reflecting patients’ daily living (45). This study demonstrated that effective rTMS treatment, as defined by changes in serum BDNF levels and depressive symptom assessments, could reverse the elevated plasma NETs levels that were observed pre-treatment. These findings further support the notion that rTMS can modulate inflammatory markers and thus positively influence the course of MDD (46). Meanwhile, regardless of treatment modality (pharmacological or non-pharmacological), post-treatment plasma NET levels were significantly reduced compared to baseline and associated with subsequent improvement in depressive symptoms following antidepressant therapy. The potential mechanisms by which NETs predict antidepressant treatment response remain unclear, and further research is needed to clarify how antidepressant drugs affect NET formation. As an initial exploratory study, our findings suggest that plasma NET markers may serve as a valuable predictor of treatment response in depression. The present study provides comprehensive evidence of the potential clinical utility of plasma NET markers in MDD, encompassing both disease diagnosis and treatment evaluation.

To investigate the possible mechanism linking peripheral NET formation to MDD, we analyzed changes in serum inflammatory factors across two cohorts. Our findings indicate that plasma NET markers are positively correlated with multiple serum inflammatory factors. Meanwhile, multivariate regression analysis revealed that plasma NET levels are associated with depressive symptoms in MDD patients, an association potentially affected by CRP-related inflammatory responses (47). The current findings reveal the role of NETs in promoting MDD development, possibly through inflammatory mechanisms. In addition, the calcarine and cuneus are key regions for visual processing, and are also implicated in multisensory integration across hearing, language, and emotion (48). Previous studies indicate that elevated fALFF values in the right calcarine may be associated with excessive attention toward negative visual information in patients with MDD (49), while significantly increased fALFF values in the right cuneus may be linked to cognitive impairment in individuals with depressive symptoms (50). In the current study, positive correlations were observed between plasma levels of NET markers and fALFF values in the calcarine and cuneus among patients with MDD. These findings suggest that peripheral NET formation may influence depressive emotion by affecting activity in specific brain regions. Consequently, both molecular and neuroimaging evidence point to the involvement of peripheral NETs in MDD.

The hippocampus plays a crucial role in mood regulation through its connectivity with emotion-related brain regions, such as the anterior cingulate cortex and amygdala, as well as via modulation of the HPA axis and neuroplasticity (51, 52). Microglia-mediated neuroinflammation may impair the formation and maturation of functional hippocampal neurogenesis, thereby contributing to the onset and progression of MDD (53, 54). Compared to control mice, CUMS mice exhibited significantly increased expression of NE and MPO in the hippocampus. Furthermore, co-localization staining revealed that NE and MPO proteins were “phagocytosed” by microglial cells in the hippocampal region, suggesting that these NETs-associated proteins may contribute to microglial activation and subsequent induction of neuroinflammation linked to depression. Moreover, plasma levels of NE-DNA, MPO-DNA, and citH3 were significantly elevated in CUMS mice compared with controls and were significantly correlated with depression severity, suggesting that peripheral NET components may be associated with the development of depression-like behaviors, potentially through their effects on the brain. Meanwhile, CUMS mice exhibited significantly elevated levels of pro-inflammatory factors compared with controls. Hence, peripheral NETs may play a role in the neuroinflammatory pathology of depression. Notably, colocalization of MPO/NE with Iba1 does not prove a peripheral origin or blood-brain barrier crossing; thus, the role of peripheral NETs can only be speculated based on the present preliminary findings. Previous studies have indicated that neurovascular dysfunction, characterized by blood-brain barrier breakdown, underlies the development of MDD (55). In CUMS mouse models, hippocampal blood-brain barrier disruption has been closely related to stress vulnerability (56, 57). It is hypothesized that peripheral NETs may appear in the brain via a compromised BBB, and their impact on brain neurons may be achieved through neuroinflammation. Besides, since neutrophils can selectively cross the blood-brain barrier and infiltrate into inflammatory regions of the hippocampus (58), NETs may be released during this process and influence neuroinflammation. The present findings only demonstrate an increase in NETs within the hippocampus of CUMS mice. Therefore, further animal experiments are required to elucidate the source of NETs in the brain—whether through blood-brain barrier leakage or alternative pathways—in the context of MDD.

To evaluate the influence of NETs on depressive-like behavior, we conducted intervention studies targeting NET formation in CUMS mice. Our findings demonstrated that disrupting NET formation, either through enzymatic digestion with DNase I or by inhibiting PAD4, significantly reduced NETs levels in both peripheral blood and hippocampal regions, decreased hippocampal expression of pro-inflammatory factors, and concurrently ameliorated depressive-like behaviors in CUMS mice. However, the therapeutic translation of PAD4 inhibition is complicated by the high expression of PAD4 in neurons, whose physiological function in the central nervous system remains poorly understood (59). The neutrophil-specific PAD4 knockout mouse represents a valuable animal model to further clarify the relationship between PAD4 inhibition and depressive-like behaviors (60, 61). Similarly, while DNase I effectively degrades NETs, its action is not specific to NETs and may affect other extracellular DNA structures (62). Despite these limitations, our study provides proof-of-concept that inhibiting NET formation may represent a viable strategy for reducing the severity of MDD. These findings underscore the necessity of developing more specific interventions that can modulate NET formation without interfering with other physiological processes.

There were several limitations in the present study. (1) The present interventional experiments in CUMS mice are preliminary and did not evaluate the impact of inhibiting NET formation on neuroinflammation. Such investigations are planned through gene knockout animal models in our following work. As a preliminary study, our current focus is on reporting the observable presence of NETs in the brain tissue of CUMS mice. Moreover, since the clinical data analysis of NETs constitutes the primary and critical content of this research, in-depth results from the animal study are not presented here. (2) Since participants were recruited from two distinct centers, collection of clinical data, such as neuropsychological assessments and brain MRI, was not fully consistent between sites. The sample collection procedures and experimental conditions were consistent across the two centers, and there were no significant differences in demographic characteristics among the three groups (**Supplementary Table 3**). However, the absence of local HCs in the second independent cohort may introduce potential center or batch effects. Accordingly, these findings should be interpreted with caution, and a fully independent cohort is warranted to validate the results of group comparisons. (3) Since all participants received antidepressant treatment, it remains unclear how plasma NET markers change during the natural progression of depressive disorders in the absence of intervention. (4) An independent cohort comprising HCs and patients with MDD is necessary to validate the diagnostic performance of plasma NET markers in distinguishing MDD from HCs. Additionally, future studies should include other psychiatric disorders, such as schizophrenia, to assess the ability of these markers to differentiate MDD from other psychiatric conditions.

## Conclusion

The present study demonstrated elevated levels of NETs in the peripheral blood of patients with MDD and in the hippocampal region of depression-like animal models compared with controls. Furthermore, these increased plasma NET markers were significantly associated with the severity of depressive symptoms and the efficacy of antidepressant treatment. Based on experimental and clinical analyses, NET formation may contribute to the pathology of MDD through inflammatory mechanisms. Although further mechanistic investigation is necessary to clarify the precise role of NETs in MDD, this study provides compelling evidence supporting NET markers as potential biomarkers for the clinical assessment of MDD. Furthermore, while inhibiting NET formation may improve depressive behaviors, achieving this effect clinically will require a targeted therapeutic approach. In light of the limitations inherent in current interventional studies, specific interventions that selectively target NET formation, including emerging approaches such as transgenic techniques or monoclonal antibodies, are expected to become a major focus of future investigations.

## Data Availability

The raw data supporting the conclusions of this article will be made available by the authors, without undue reservation.
