# Supplementary material for: Exploring potential value of neutrophil extracellular traps in major depressive disorder
Source: Front Immunol. 2026 May 12;17:1818375. doi: 10.3389/fimmu.2026.1818375 (PMC13201104; doi:10.3389/fimmu.2026.1818375)
Supplement: Supplementary file 1 [file DataSheet1.docx]

**Supplementary method**

#### Operationalization of Major Depressive Disorder (MDD) According to DSM-V

MDD was operationalized strictly according to DSM-V criteria as follows:

**A. Criterion A** (≥5 of the following symptoms within the same 2-week period, representing a change from previous functioning; at least one symptom is either (1) or (2)):

1) Depressed mood (subjective or observed);

2) Markedly diminished interest or pleasure (anhedonia);

3) Significant weight/appetite change;

4) Insomnia or hypersomnia;

5) Psychomotor agitation or retardation;

6) Fatigue or loss of energy;

7) Feelings of worthlessness or excessive guilt;

8) Diminished ability to think or concentrate, or indecisiveness;

9) Recurrent thoughts of death, suicidal ideation, or attempt.

**B. Exclusion criteria**:

1) Symptoms are not attributable to substance use (e.g., drug, medication) or another medical condition (e.g., hypothyroidism);

2) No history of manic or hypomanic episode (to rule out bipolar disorder).

**C. Clinically significant distress or impairment in social, occupational, or other important domains of functioning.**

Furthermore, given that anxiety symptoms commonly co-occur with MDD, the following strategy was employed for further differentiation, with the exception of symptom content.

1) Anxiety symptoms present only during depressive episodes and parallel depression severity, which considered associated features of MDD (no separate anxiety disorder).

2) Anxiety symptoms persisting after depression remission, which diagnosed as comorbid anxiety disorder.

#### Image acquisition

A 3.0T MR scanner is used in the present study. The data of high-resolution T1-weighted images (T1WI) and resting-state functional MRI (rs-fMRI) was collected. None of subjects had excessive motion artifacts (≥ 2 mm translational or ≥ 2° rotational movements) or incomplete image coverage.

The scanning parameters were as follows: Protocol name = EPI; Repetition time = 1500 ms; Echo time = 31 ms; Flip angle = 70°; Field of view = 211 mm × 211 mm; Matrix = 88 × 88; Thickness (gap) = 2.4 mm (0); Slice number = 60.

#### Rs-fMRI data preprocessing

Rs-fMRI data were preprocessed using SPM8 and the Data Processing Assistant for Resting-State fMRI (DPARSF, <http://www.restfmri.net/forum/dparsf>). The initial 10 functional volumes were discarded for scanner stabilization and participant adaption. The remaining images were corrected for timing differences and motion effects. The individual structural images (T1WI) were co-registered to mean functional images after motion correction using linear transformation. Motion corrected functional volumes were spatially normalized to the Montreal Neurological Institute space using DARTEL toolbox and resampled to a voxel size of 3 mm × 3 mm × 3 mm. To further reduce the effects of confounding factors, Friston 24 motion parameters, white matter signal, and cerebrospinal fluid signal were removed from the data via linear regression. Then, smoothing with a 6-mm full-width at half-maximum kernel and linear detrending were performed.

#### Fractional amplitude of low-frequency fluctuations (fALFF) analyses

DPARSF software was used for the present analyses [[4](#_ENREF_4)]. The fALFF reflects the strength of regional slow-wave brain activity. The smoothed functional images derived from the first preprocessing steps were subjected to linear detrending and temporal filtering (0.01–0.08 Hz) to remove undesired components. The time series of each voxel was transformed into the frequency domain through a Fourier transform. The sum of the magnitude of the spectrum between 0.01 and 0.08 Hz was divided by the sum of the entire acquired frequency band to obtain the fALFF.

#### Statistical analysis of MRI data

REST software was used for the analysis of MRI data across groups and group differences in whole-brain fALFF values. Two-sample t-test was used for assess the between-group differences in fALFF values across DD and HC groups, with age, sex, body mass index, and years of education as covariates. The results were considered significant differences at a corrected p < 0.05 and cluster size > 18 voxels. Multiple comparison correction was performed using the Alphasim multiple comparison correction. Mean fALFF values of clusters exhibiting significant differences between groups were extracted.

**Behavioural tests**

The sucrose preference test was used to evaluate the mice's preference for sugar and to assess anhedonia. Briefly, mice were habituated to a 1% (w/v) sucrose solution by providing a single bottle for 3 days. Following habituation, baseline sucrose preference was measured over 24 hours by offering both tap water and sucrose solution simultaneously. Subsequently, a 24‑hour sucrose preference test was conducted where two identical bottles-one containing tap water and the other containing sucrose solution-were provided. The positions of the bottles were switched every 6 hours, and intake from both bottles was measured. Sucrose preference was calculated as: percentage preference = (sucrose intake / total intake) × 100. All tests were performed by an experimenter blinded to the treatment groups.

Additionally, the open field test was conducted to evaluate locomotor and exploratory behaviors. The open-field arena was divided into 16 equal squares. Each mouse was placed in the center area and allowed to habituate for 2 minutes. Over the subsequent 3 minutes, the total distance traveled, time spent in the center, and number of center crossings were recorded. These tests were also carried out by an observer blinded to the animals’ treatment assignments.

**Immunofluorescence imaging analysis**

Mice were deeply anesthetized with isoflurane and transcardially perfused with ice-cold PBS (pH 7.4), followed by 4% paraformaldehyde (PFA). The dissected brains were post-fixed in 4% PFA at 4°C for 24–48 h, rinsed, dehydrated through a graded ethanol series, cleared in xylene, and embedded in paraffin using an automated tissue processor (JJ-12J, Wuhan Junjie Electronics). Serial sections (4–5 μm thick) were cut, mounted on poly-L-lysine-coated slides, dried, and baked at 60°C for 2 h.

For immunofluorescence, sections were deparaffinized, rehydrated, and permeabilized with 0.3% Triton X-100 in PBS (10 min at room temperature). After blocking with 10% normal donkey serum containing 0.1% Triton X-100 (60 min at room temperature), sections were incubated overnight at 4°C with the following primary antibodies: anti-MPO (1:50, ab25989, Abcam), anti-NE (1:500, ab314916, Abcam), and anti-Iba1 (1:250, 019-19741, Wako Pure Chemicals). After washing with PBS (three times, 5 min each), sections were incubated with fluorescent secondary antibodies — donkey anti-mouse IgG conjugated to Alexa Fluor 594 and donkey anti-rabbit IgG conjugated to Alexa Fluor 488 (each 1:500, Abcam) — for 50 min at room temperature in the dark. Following another series of PBS washes (three times, 5 min each), nuclei were counterstained with DAPI (Servicebio G1012) for 10 min, rinsed, and mounted with anti-fade mounting medium. Images were acquired using a fluorescence/confocal slide scanner (3DHISTECH Pannoramic MIDI) with standard DAPI, FITC, and TRITC filter sets.

**Supplementary Table 1. The ROC analysis of three NETs markers for distingushing DD from HCs in Cohort 1.**

|  | 95% CI | specificity | sensitivity |
| --- | --- | --- | --- |
| NE-DNA | 0.626 - 0.825 | 77.78% | 68.42% |
| MPO-DNA | 0.657 - 0.849 | 53.33% | 89.47% |
| citH3 | 0.748 - 0.915 | 68.89% | 89.47% |

**Supplementary Table 2. Correlation analyses between plasma NETs markers and clinical features in MDD patients in Cohort 1 (original data of r-value).**

| r-value | NE-DNA | MPO-DNA | citH3 | BDNF | CRP | IL-6 | TNF-α | HAMD-24 | HAMA | SDS | SAS | SHAPS |
| --- | --- | --- | --- | --- | --- | --- | --- | --- | --- | --- | --- | --- |
| NE-DNA | 1 | 0.44 | 0.398 | 0.042 | 0.25 | 0.286 | 0.039 | 0.558 | 0.28 | 0.261 | 0.251 | 0.325 |
| MPO-DNA | 0.44 | 1 | 0.385 | 0.124 | 0.24 | 0.232 | 0.061 | 0.566 | 0.492 | 0.242 | 0.324 | 0.141 |
| citH3 | 0.398 | 0.385 | 1 | -0.012 | 0.615 | 0.362 | -0.23 | 0.721 | 0.518 | 0.354 | 0.55 | 0.418 |
| BDNF | 0.042 | 0.124 | -0.012 | 1 | -0.275 | -0.339 | 0.103 | -0.08 | -0.142 | -0.036 | -0.024 | -0.174 |
| CRP | 0.25 | 0.24 | 0.615 | -0.275 | 1 | 0.257 | -0.433 | 0.648 | 0.54 | 0.387 | 0.526 | 0.468 |
| IL-6 | 0.286 | 0.232 | 0.362 | -0.339 | 0.257 | 1 | -0.133 | 0.397 | 0.381 | 0.304 | 0.403 | 0.254 |
| TNF-α | 0.039 | 0.061 | -0.23 | 0.103 | -0.433 | -0.133 | 1 | -0.229 | -0.214 | -0.199 | -0.309 | -0.4 |
| HAMD-24 | 0.558 | 0.566 | 0.721 | -0.08 | 0.648 | 0.397 | -0.229 | 1 | 0.708 | 0.611 | 0.526 | 0.582 |
| HAMA | 0.28 | 0.492 | 0.518 | -0.142 | 0.54 | 0.381 | -0.214 | 0.708 | 1 | 0.641 | 0.568 | 0.556 |
| SDS | 0.261 | 0.242 | 0.354 | -0.036 | 0.387 | 0.304 | -0.199 | 0.611 | 0.641 | 1 | 0.573 | 0.506 |
| SAS | 0.251 | 0.324 | 0.55 | -0.024 | 0.526 | 0.403 | -0.309 | 0.526 | 0.568 | 0.573 | 1 | 0.335 |
| SHAPS | 0.384 | 0.141 | 0.418 | -0.174 | 0.468 | 0.254 | -0.4 | 0.582 | 0.556 | 0.506 | 0.335 | 1 |

**Supplementary Table 3. Correlation analyses between plasma NETs markers and clinical features in MDD patients in Cohort 2 (original data of r-value).**

| r-value | NE-DNA | MPO-DNA | citH3 | BDNF | CRP | IL-6 | TNF-α | HAMD-24 | SDS | SAS |
| --- | --- | --- | --- | --- | --- | --- | --- | --- | --- | --- |
| NE-DNA | 1 | 0.311 | 0.289 | -0.296 | 0.256 | 0.263 | 0.020 | 0.384 | 0.035 | -0.013 |
| MPO-DNA | 0.311 | 1 | 0.286 | -0.302 | 0.319 | 0.316 | 0.174 | 0.319 | 0.332 | 0.137 |
| citH3 | 0.289 | 0.286 | 1 | -0.225 | 0.301 | 0.369 | 0.316 | 0.482 | 0.328 | 0.320 |
| BDNF | -0.296 | -0.302 | -0.225 | 1 | -0.247 | -0.26 | -0.173 | -0.304 | -0.043 | -0.117 |
| CRP | 0.256 | 0.319 | 0.301 | -0.247 | 1 | 0.353 | 0.114 | 0.564 | -0.012 | 0.204 |
| IL-6 | 0.263 | 0.316 | 0.369 | -0.26 | 0.353 | 1 | 0.301 | 0.453 | 0.192 | 0.186 |
| TNF-α | 0.020 | 0.174 | 0.316 | -0.173 | 0.114 | 0.301 | 1 | 0.298 | 0.133 | 0.077 |
| HAMD-24 | 0.384 | 0.319 | 0.482 | -0.304 | 0.564 | 0.453 | 0.298 | 1 | 0.375 | 0.123 |
| SDS | 0.035 | 0.332 | 0.328 | -0.043 | -0.012 | 0.192 | 0.133 | 0.375 | 1 | 0.32 |
| SAS | -0.013 | 0.137 | 0.320 | -0.117 | 0.204 | 0.186 | 0.077 | 0.123 | 0.32 | 1 |

**Supplementary Table 4. Assessments of depressive-like behaviors in CUMS and control mice.**

|  | CUMS (n=6) | Control (n=6) | P-value |
| --- | --- | --- | --- |
| Open Field Test |  |  |  |
| Total distance traveled (mm) | 22588 ± 3706 | 34040 ± 5462 | 0.002 |
| Immobility time in the all district (s) | 74.88 ± 15.96 | 31.05 ± 8.84 | <0.001 |
| Distance traveled in the center (mm) | 17513 ± 377.84 | 33672 ± 1084 | 0.006 |
| Time spent in the center (s) | 9.70 ± 5.28 | 18.88 ± 7.86 | 0.039 |
| Sucrose preference test |  |  |  |
| Percentage of sucrose preference | 0.28 ± 0.08 | 0.87 ± 0.06 | <0.001 |

CUMS, chronic unpredictable mild stress.


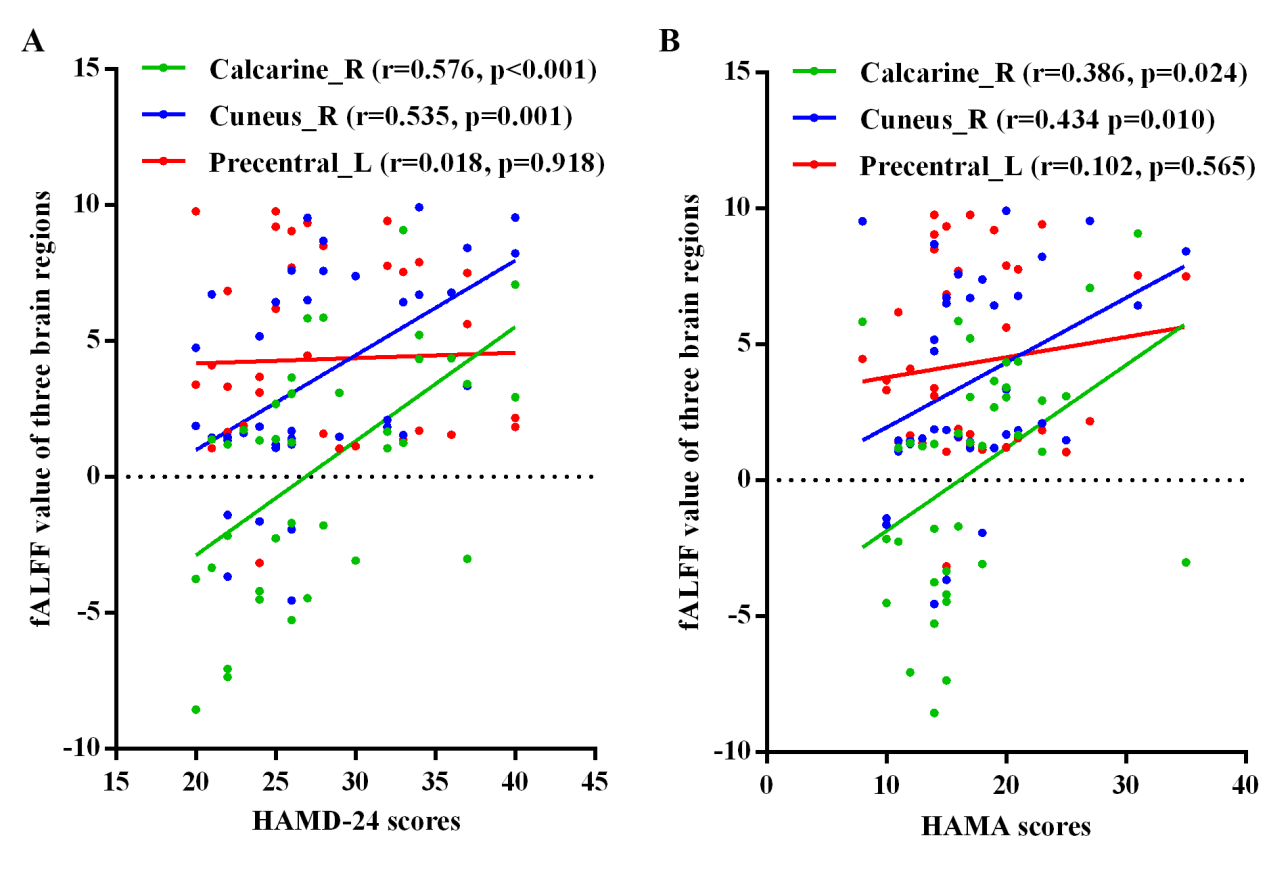


**Supplementary Figure 1. Association between fALFF value of three brain regions and HAMD-24 (A) and HAMA (B) scores at baseline.**

Age, sex, body mass index, and education years were controlled.

R, right; L, left.


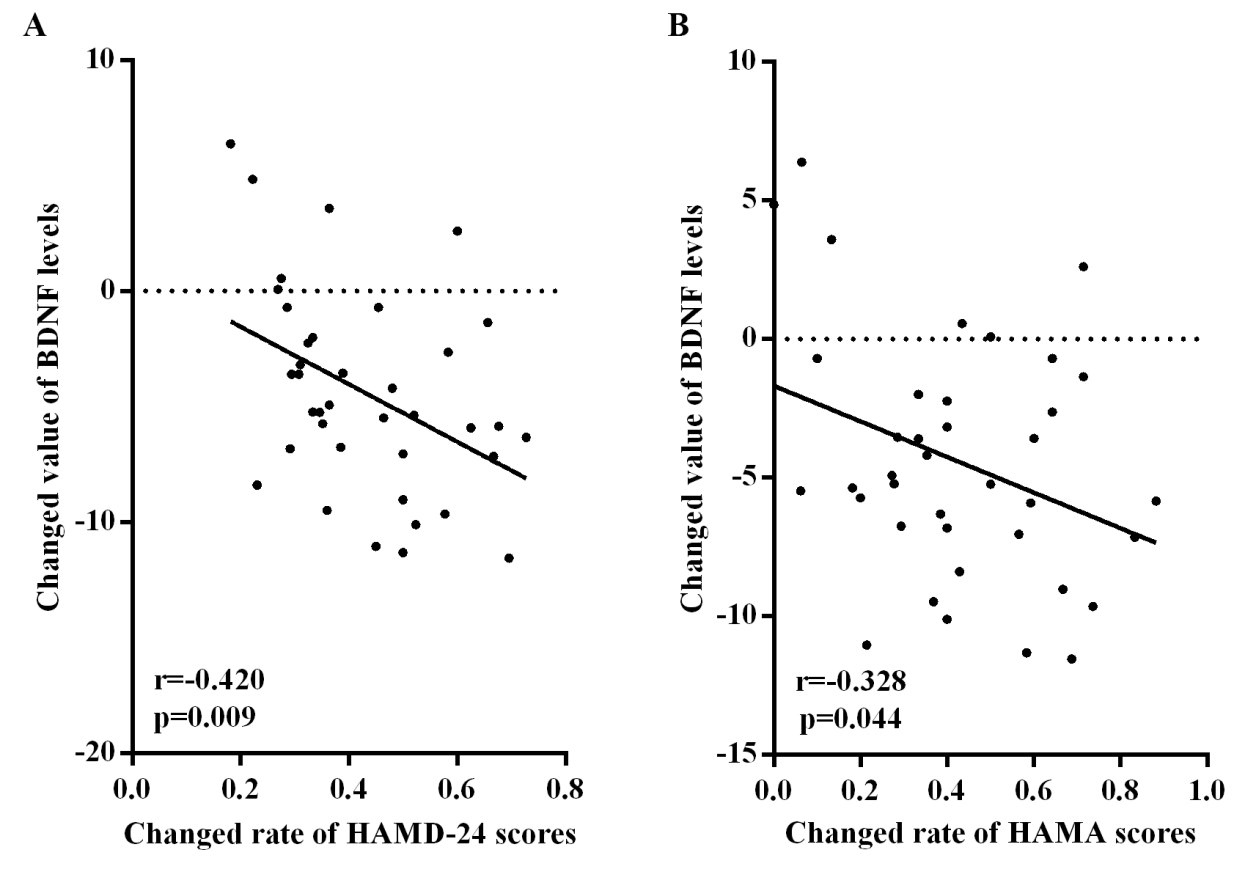


**Supplementary Figure 2. Association between changed value of BDNF and changed rate of HAMD-24 (A) and HAMA (B) scores.**

Age, sex, body mass index, and education years were controlled.


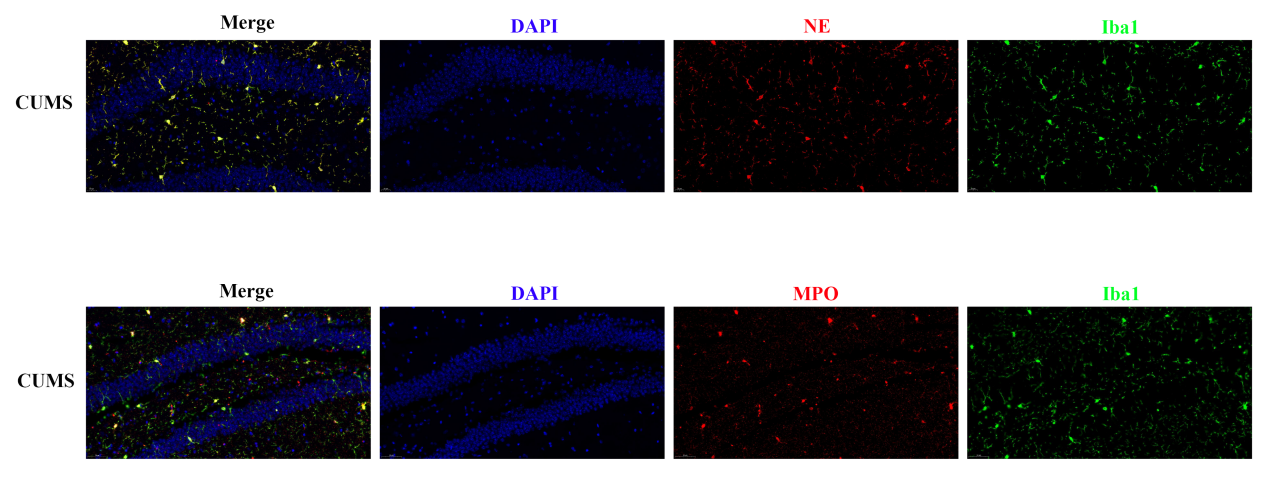


**Supplementary Figure 3. Association between NE/MPO and microglial cell in CUMS animal models.**
